# Supplementary material for: Structure and energy transfer of a far-red–absorbing euglenophyte PSI–LhcE–LhcbM supercomplex
Source: Nat Commun. 2026 Feb 27;17:3273. doi: 10.1038/s41467-026-70067-1 (PMC13066549; doi:10.1038/s41467-026-70067-1)
Supplement: Supplementary file 1 — Supplementary Information [file 41467_2026_70067_MOESM1_ESM.pdf]

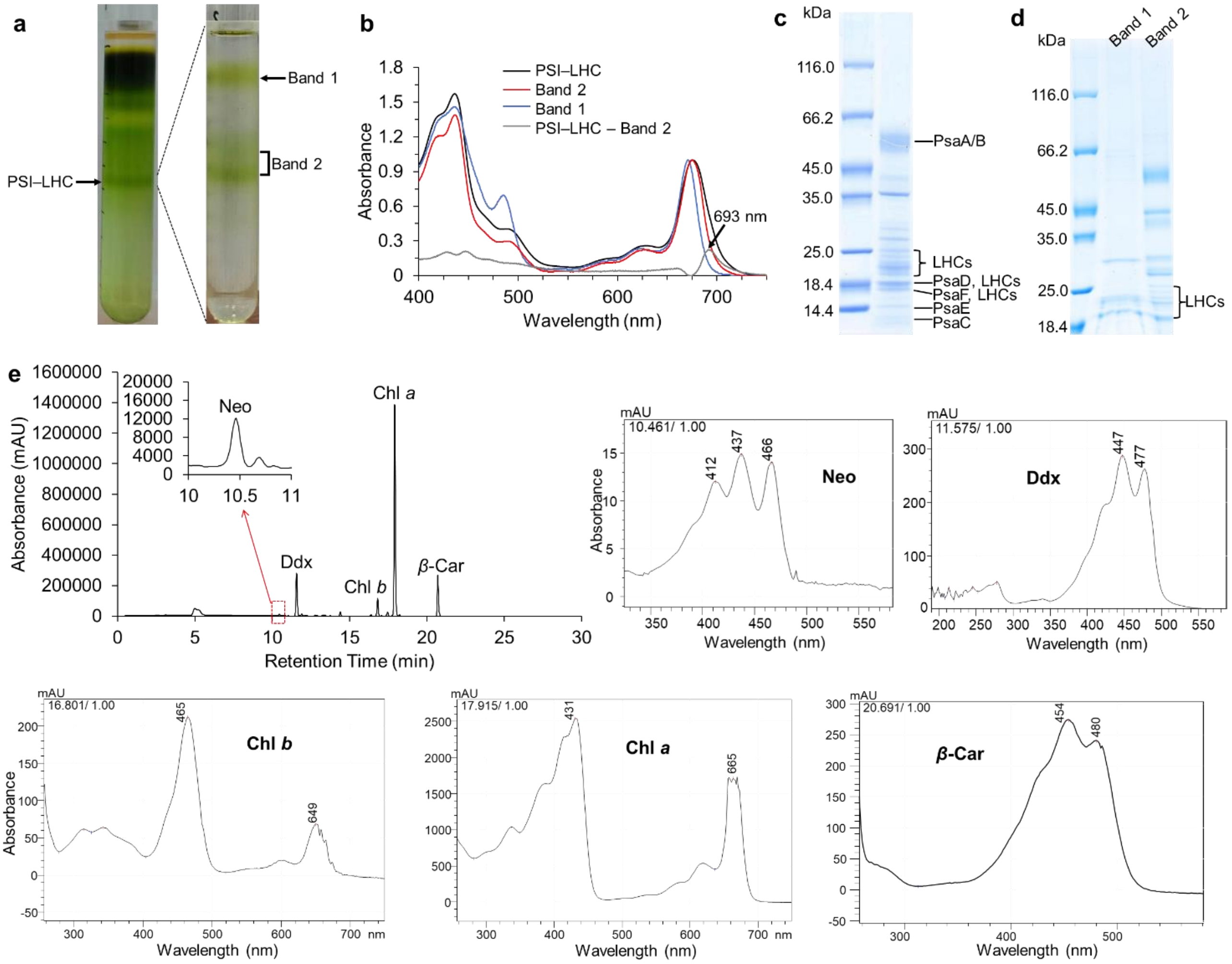

Supplementary Figure 1. Preparation and characterization of PSI–LhcE–LhcbM from euglenophyte *E. gracilis*.

a, Isolation of the *E. gracilis* PSI–LhcE–LhcbM supercomplex using sucrose density gradient by ultracentrifugation (left tube). The LHCs were dissociated from the PSI–LhcE–LhcbM by a second sucrose density gradient ultracentrifugation (right tube). **b**, Room-temperature absorption spectra of the PSI–LhcE–LhcbM band, Band 1, and Band 2 in panel **a**. The differential absorption spectrum between PSI–LhcE–LhcbM and Band 2 exhibits a far-red absorption peak at 693 nm. **c**, SDS-PAGE analysis of the PSI–LhcE–LhcbM supercomplex. The protein composition of the bands was indicated based on the mass spectrometry analysis. **d**, SDS-PAGE analysis of Band 1 and Band 2 in panel **a**. **e**, Pigment analysis of the *E. gracilis* PSI–LhcE–LhcbM supercomplex by high performance liquid chromatography, recorded at 445 nm. Based on the characteristic absorption spectrum of each peak fraction, neoxanthin (Neo), diadinoxanthin (Ddx), chlorophyll *b* (Chl *b*), chlorophyll *a* (Chl *a*) and *β*-carotene (*β*-Car) were identified. The peak of neoxanthin is enlarged. Absorption spectra of these peaks are shown. These experiments were performed for more than three times, and the same results were obtained reproducibly. Source data are provided as a Source Data file.

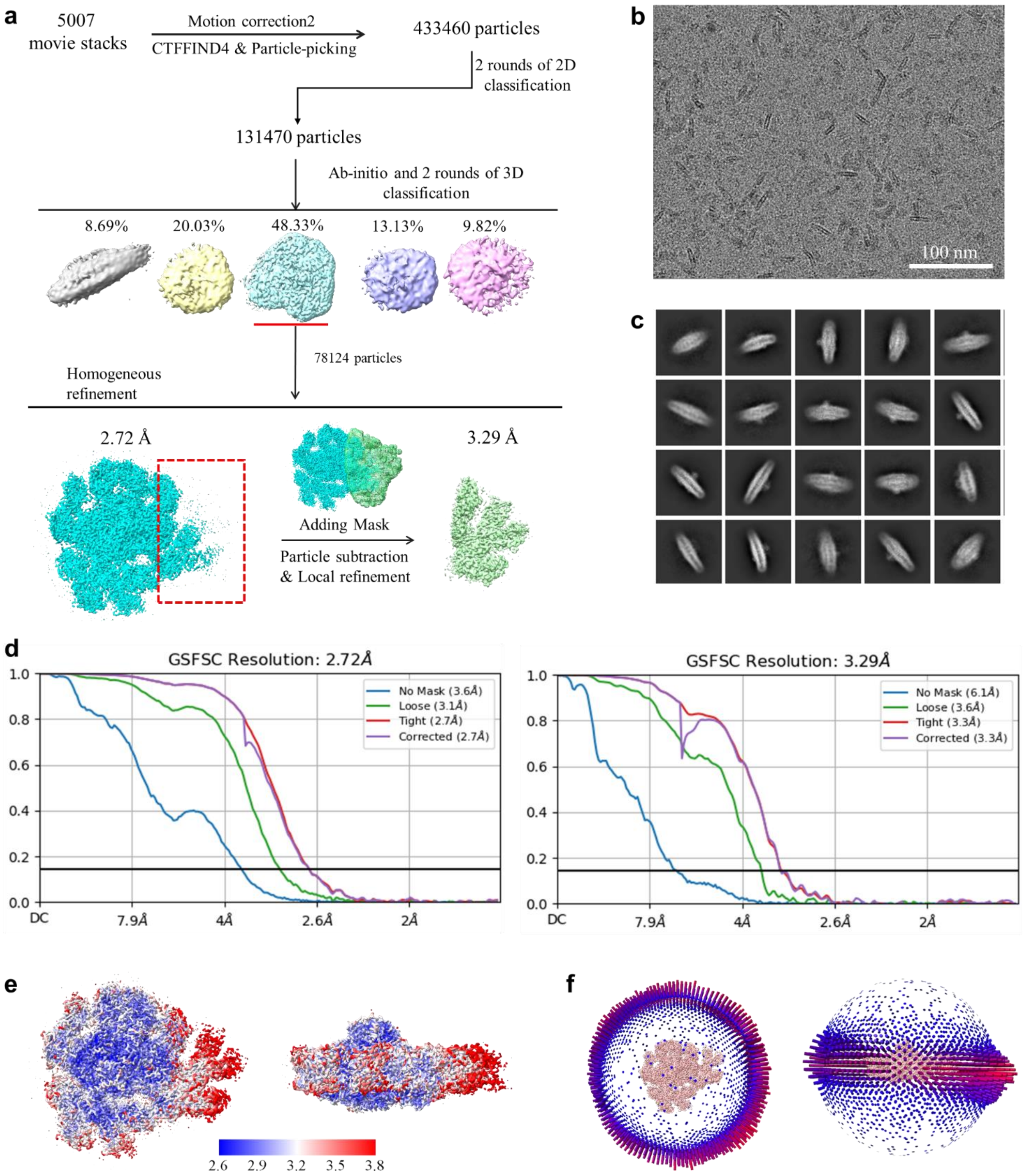

Supplementary Figure 2. Cryo-EM data processing for the euglenophyte PSI–LhcE–LhcbM supercomplex.

a, Schematic flowchart for the cryo-EM data processing. **b**, A representative cryo-EM micrograph of the euglenophyte PSI–LhcE–LhcbM supercomplex. **c**, Representative 2D classes of the euglenophyte PSI–LhcE–LhcbM supercomplex. The box size is 417 Å. **d**, The gold standard Fourier shell correlation (FSC) curves for estimation of the resolution of the overall density map (left panel) and local density map (right panel) with criterion of 0.143. **e**, Local resolution distributions of the cryo-EM map estimated by ResMap. **f**, Angular distribution of particles used for reconstruction of the final density map.

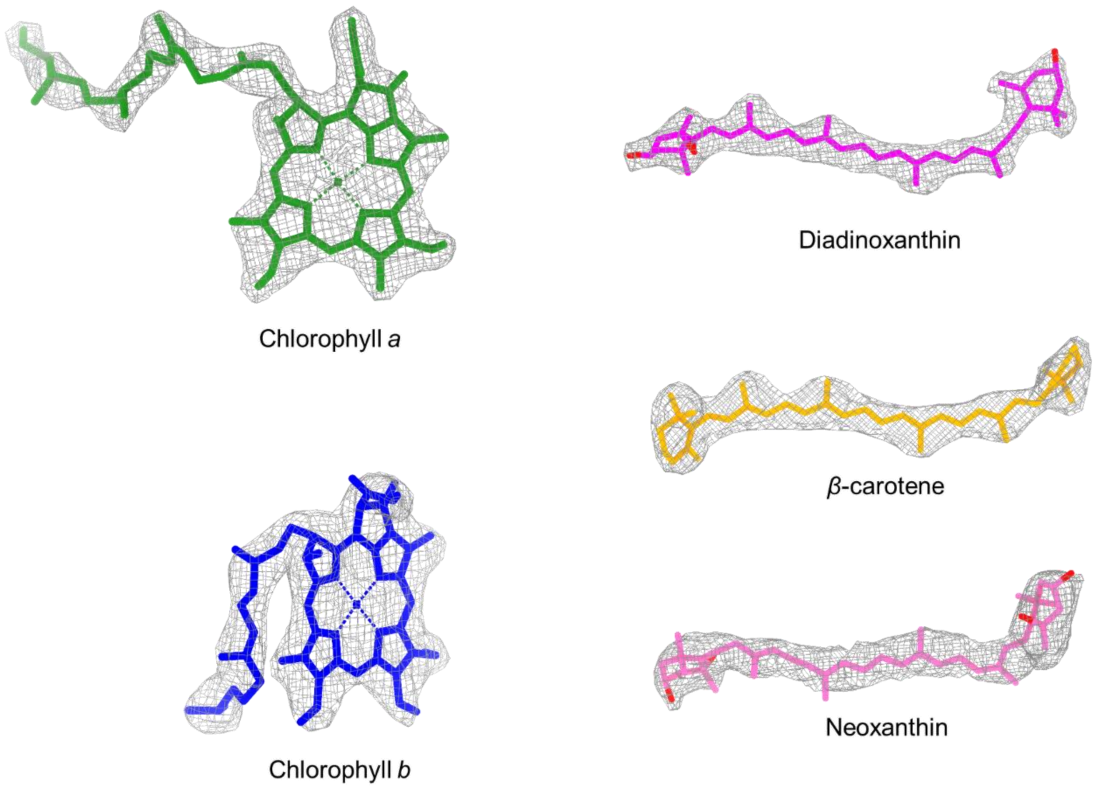

Supplementary Figure 3. Cryo-EM density maps and structures of pigment molecules in the euglenophyte PSI–LhcE–LhcbM supercomplex.

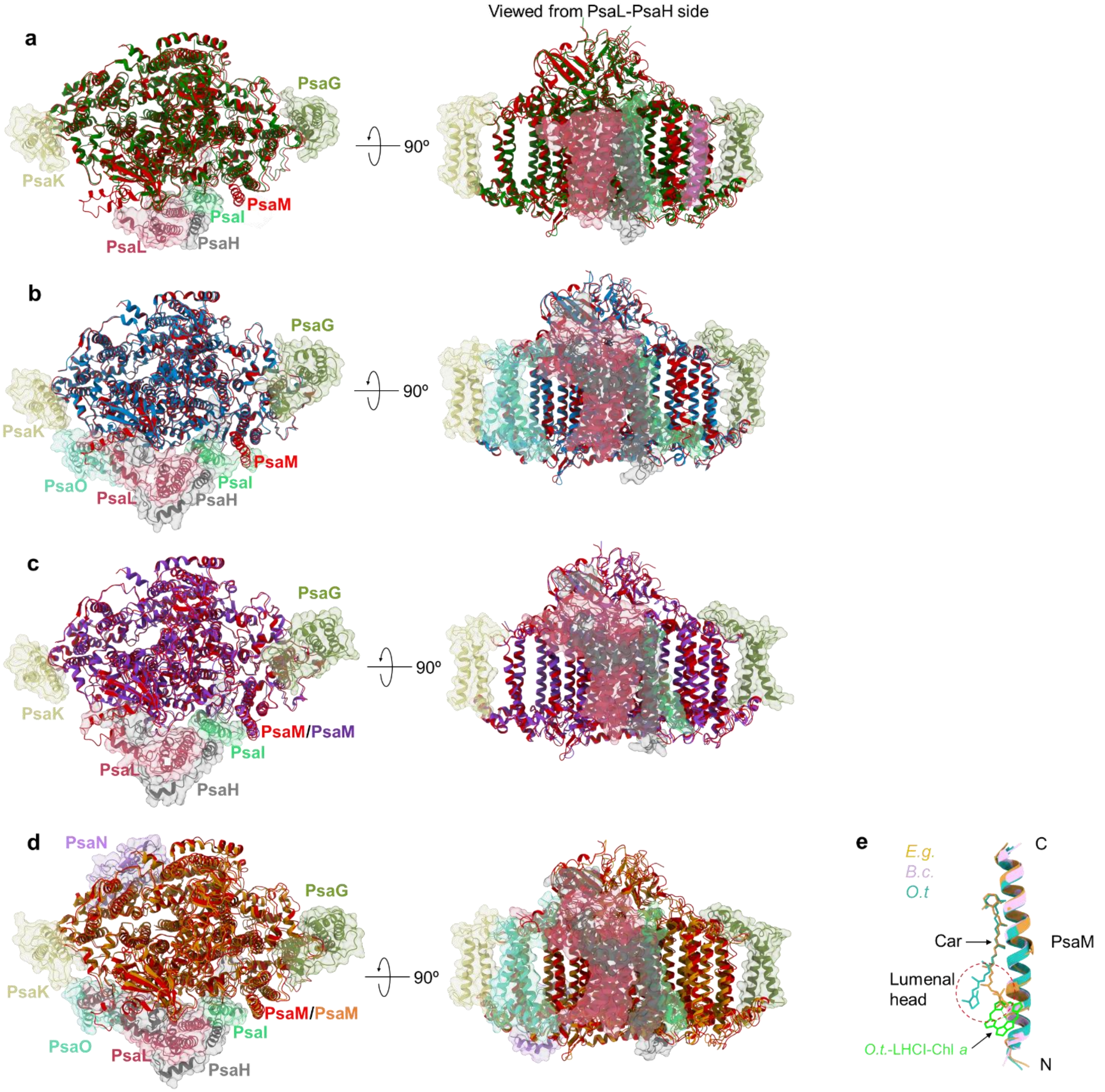

Supplementary Figure 4. Comparison of the PSI cores of euglenophyte and green algae.
Superposition of *E. gracilis* PSI core (red) with *Chlamydomonas reinhardtii* PSI core (green, PDB: 6IJO) (**a**), *Dunaliella Salina* PSI core (blue, PDB: 6SL5) (**b**), *Bryopsis corticulans* PSI core (purple, PDB: 6IGZ) (**c**), and *Ostreococcus tauri* PSI core (orange, PDB: 7YCA) (**d**). PsaG, PsaK, PsaH, PsaI, PsaL, PsaO, and PsaN, which are absent in euglenophyte PSI core, are indicated. PsaM is labeled. **e**, The orientation of Car in PsaM, and comparison with those in PsaM of green algae *Bryopsis corticulans* (*B.c.*) and *Ostreococcus tauri* (*O.t.*). The swing on the lumenal head group of the Car is indicated by red circle.

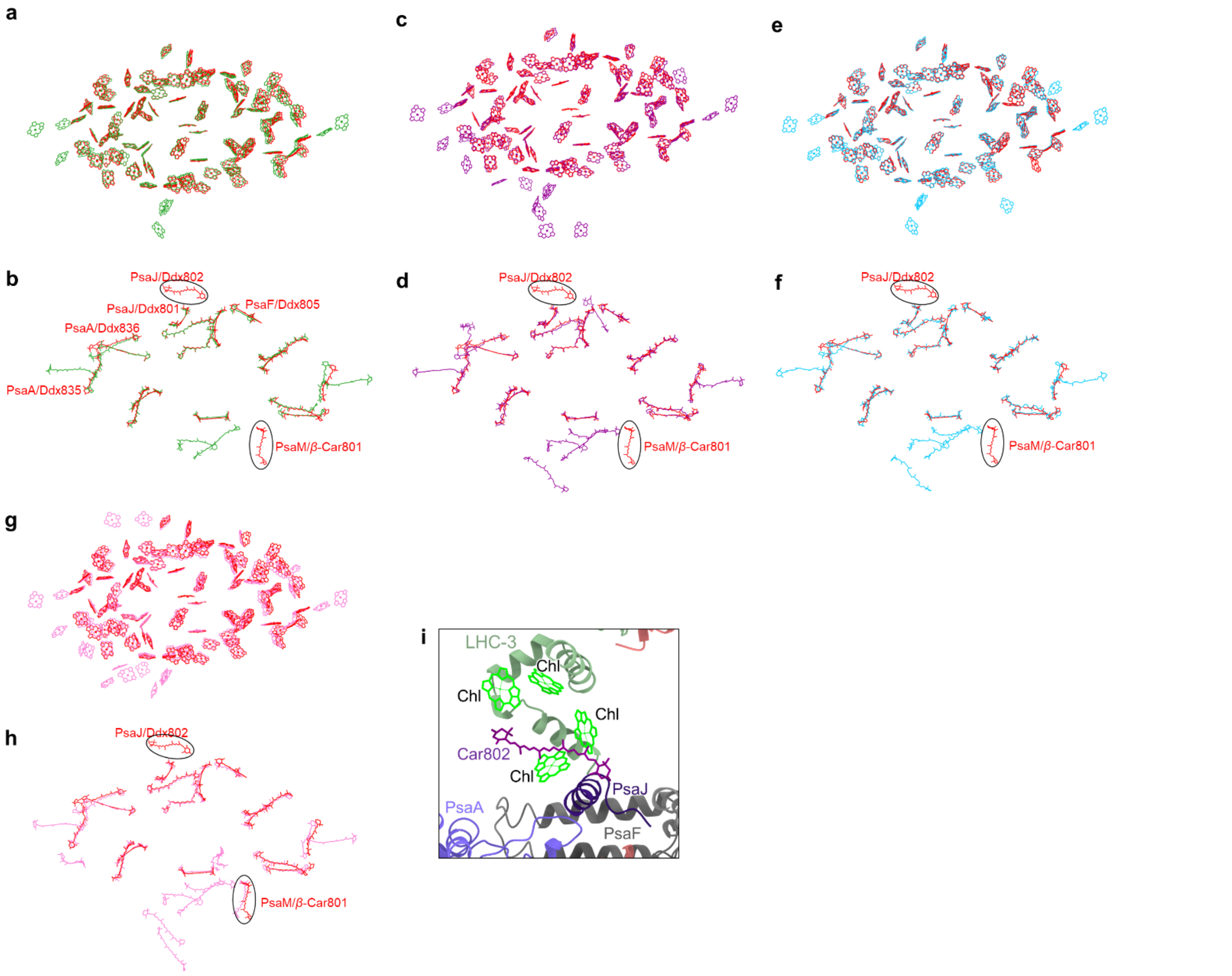

Supplementary Figure 5. Comparation of pigment arrangements in euglenophyte PSI core with those in marine green-algal PSI core. Superposition of the Chl sites (left panel) and carotenoid sites (right panel) in euglenophyte PSI core (red) with those in *Chlamydomonas reinhardtii* PSI core (green, PDB: 6IJO) (**a, b**), *Dunaliella Salina* PSI core (blue, PDB: 6SL5) (**c, d**), *Bryopsis corticulans* PSI core (purple, PDB: 6IGZ) (**e, f**), and *Ostreococcus tauri* PSI core (orange, PDB: 7YCA) (**g, h**). The additional PsaJ/Ddx802 and the PsaM/β-Car801 are indicated by black ovals. The β-Car sites which are substituted by Ddx are labeled. All panels are viewed from the stromal side. **i**, The location of the additional Car802 in PsaJ, which is absent in green algae. The surrounding chlorophylls are indicated.

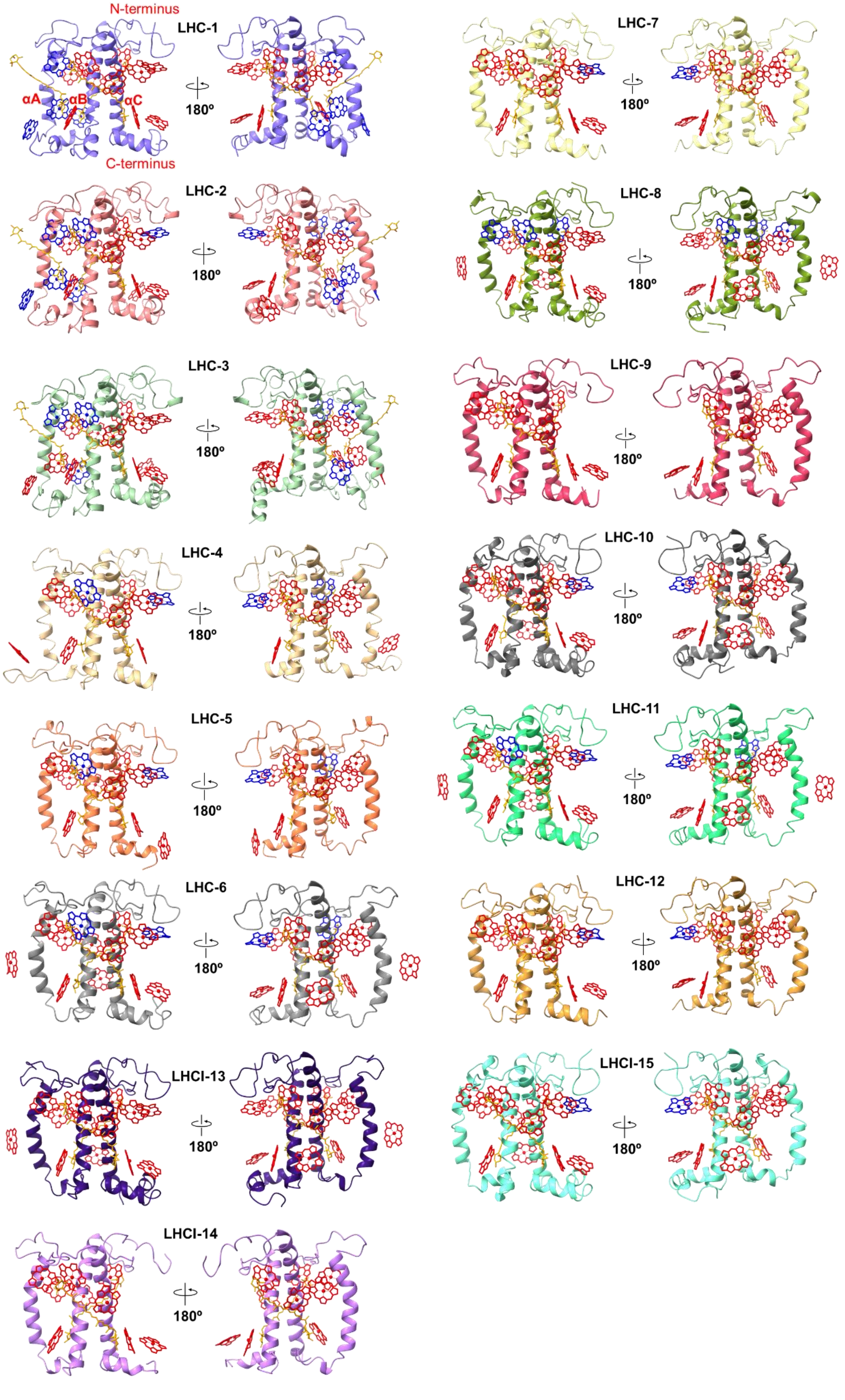

Supplementary Figure 6. Structures of individual 15 LHCs subunits of euglenophyte PSI–LhcE–LhcbM. Chl *a*, Chl *b* and Cars are colored in red, blue and orange, respectively. The phytol chains of Chls are omitted.

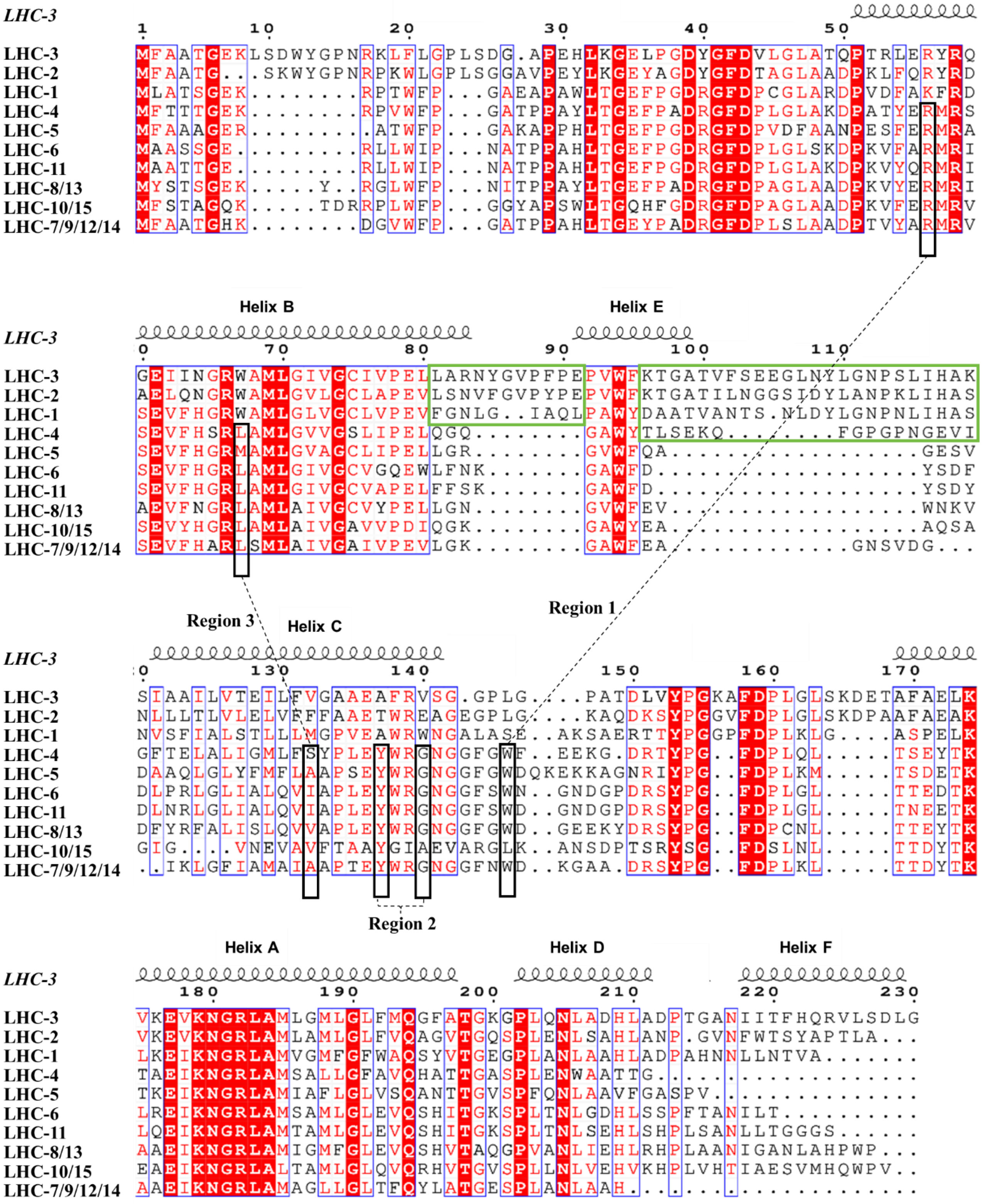

Supplementary Figure 7. Sequence alignment of LHCs from the *E. gracilis* PSI–LhcE–LhcbM.
The secondary structure is shown above the sequences. Fully conserved residues are shaded in red, and similar amino acids are highlighted by blue frames. The extended BE loops and CE loops are highlighted by green boxes.

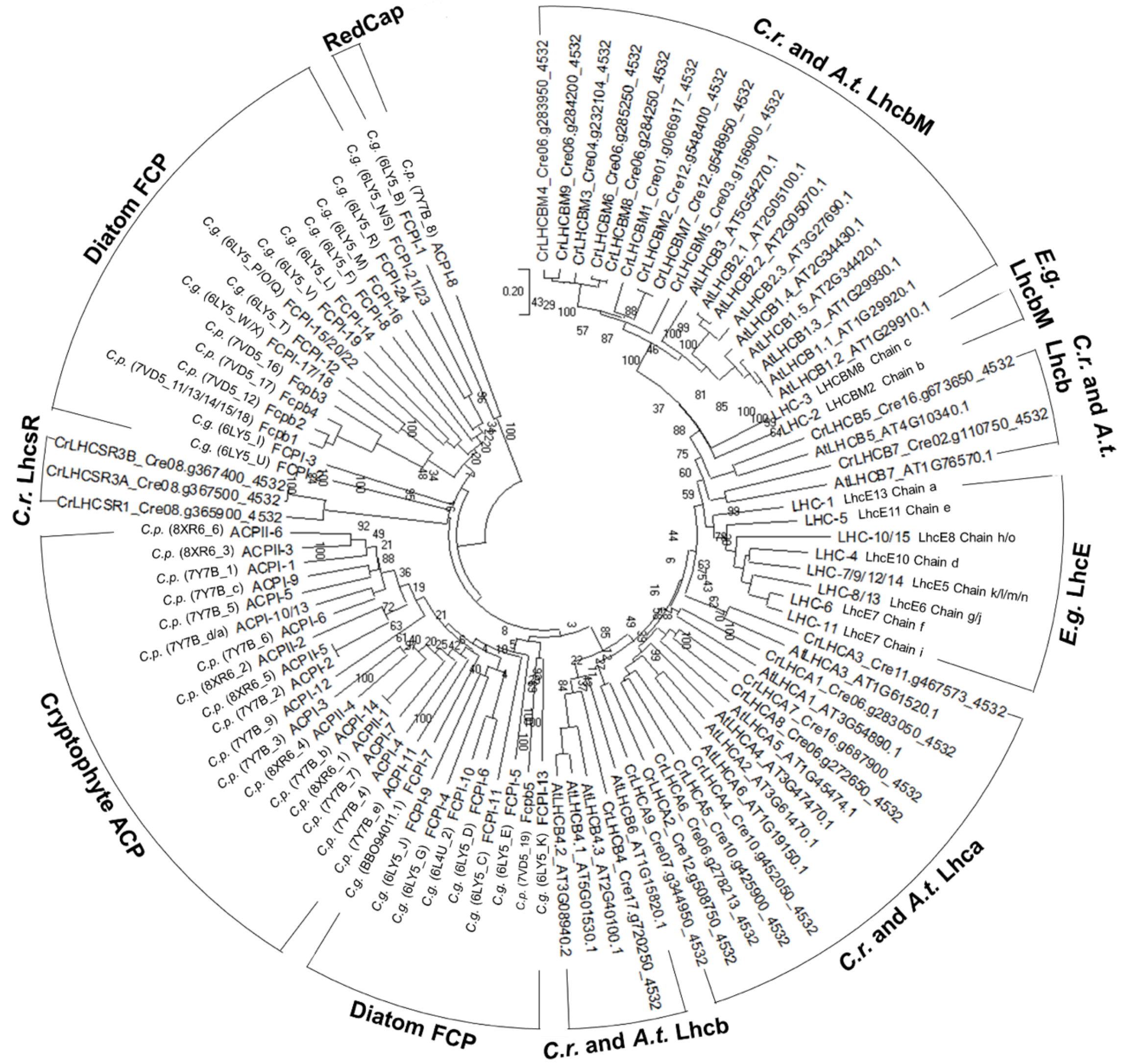

Supplementary Figure 8. Phylogenetic tree of LHCs from *E. gracilis* (*E.g.*) PSI–LhcE–LhcbM, green algae *Chlamydomonas reinhardtii* (*C.r.*), land plant *Arabidopsis thaliana* (*A.t.*), cryptophyte *Chroomonas placoidea* (*C.p.*), diatom *Chaetoceros gracilis* (*C.g.*). The Neighbor-Joining tree was based on amino acid sequences of LHCs. The tree was built using 608 amino acid residues, and a bootstrap test (1000 replicates) was conducted. Lhc family and Chain ID of each *E.g.* LHC is labeled. *C.r.* LHCs sequences [1] and *A.t.* LHCs sequences [2] of were retrieved from the Phytozome database (https://phytozome.jgi.doe.gov/) [3]. The PDB ID and Chain ID of diatom and cryptophyte LHCs are indicated in brackets in the form of “PDB ID_Chain ID”.

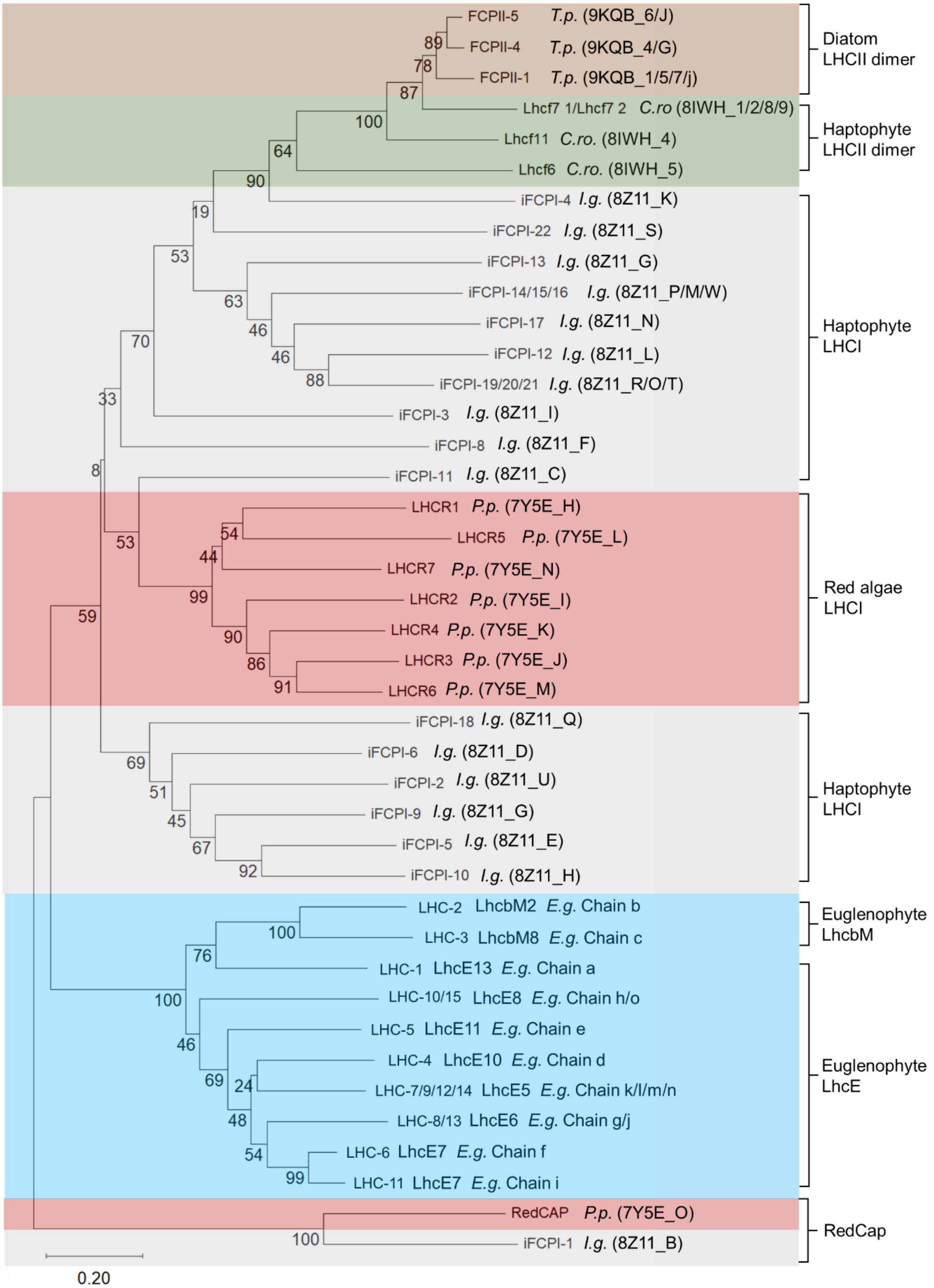

Supplementary Figure 9. Phylogenetic tree of LHCs from *E. gracilis* (*E.g.*) PSI–LhcE–LhcbM (blue), LHCIs from red alga *Porphyridium purpureum* (*P.p.*) PSI–LHCI (pink), LHCIs from haptophyte *I. galbana* (*I.g.*) PSI–LHCI (grey), LHCII dimers from haptophyte *Chyrostila roscoffensis (C.ro.)* PSII–LHCII (green), and LHCII dimers from diatom *Thalassiosira pseudonana* (*T.p.*) PSII–LHCII (brown). The Neighbor-Joining tree was based on amino acid sequences of LHCs. The tree was built using 320 amino acid residues, and a bootstrap test (1000 replicates) was conducted. Lhc family and Chain ID of each *E.g* LHC is labeled. The PDB ID and Chain ID of other LHCs are indicated in brackets in the form of “PDB ID_Chain ID”.

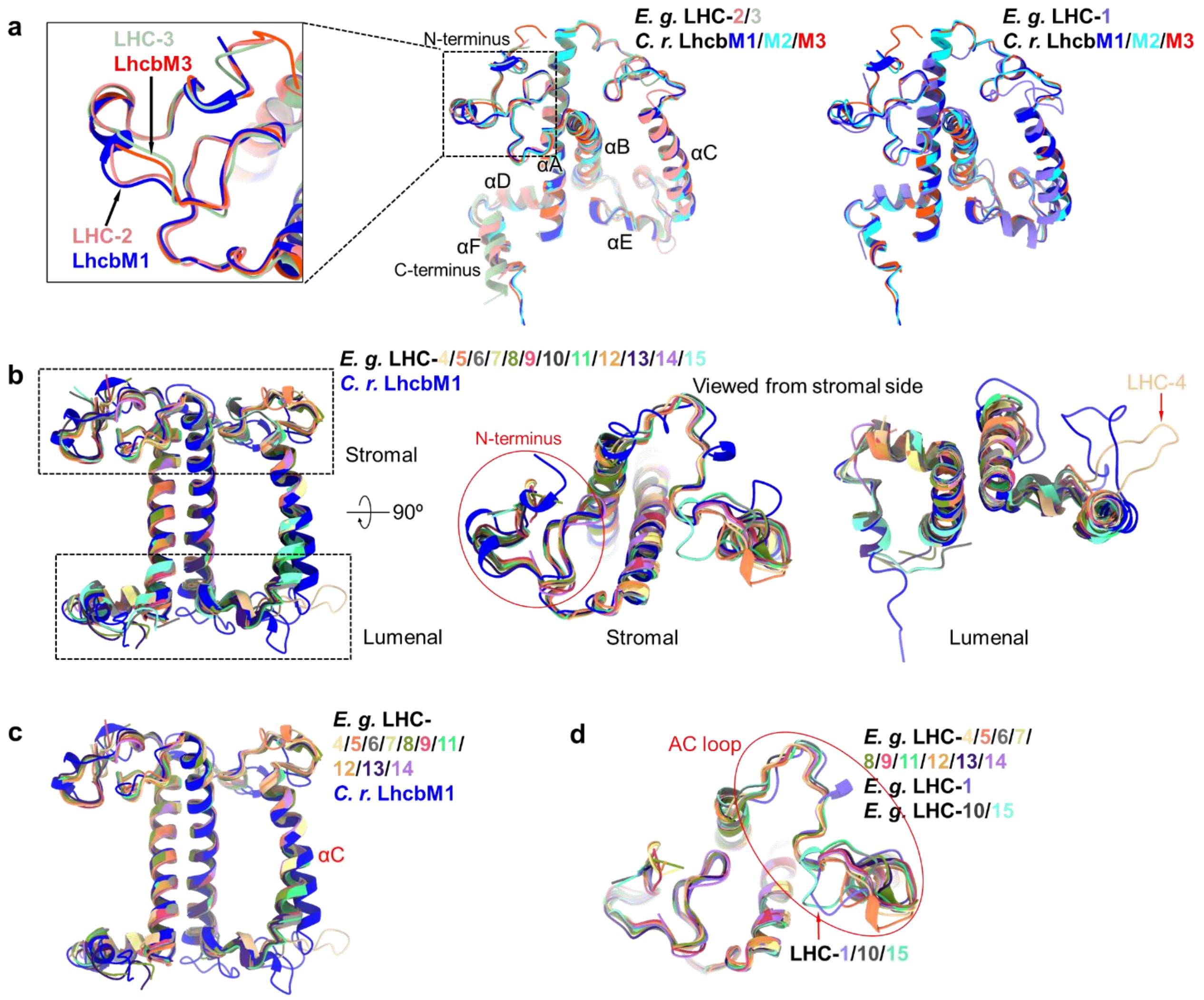

Supplementary Figure 10. Structural comparison of the euglenophyte LHCs with LhcbMs from green-algal LHCII trimer (PDB:6KAD). **a**, Structural comparison of euglenophyte LhcE-type LHC-1 and LhcbM-type LHC-2/3 with *C. reinhardtii* (*C. r.*) LhcbM1/M2/M3. **b**, Structural comparison of euglenophyte LhcE-type LHC-(4-15) with *C. reinhardtii* (*C. r.*) LhcbM1. **c**, Structural comparison of euglenophyte LHCs of LhcE-type LHC dimers with *C. reinhardtii* (*C.r.*) LhcbM1. **d**, Structural comparison of euglenophyte LHCs of LhcE-type LHC dimers with euglenophyte LHC monomers.

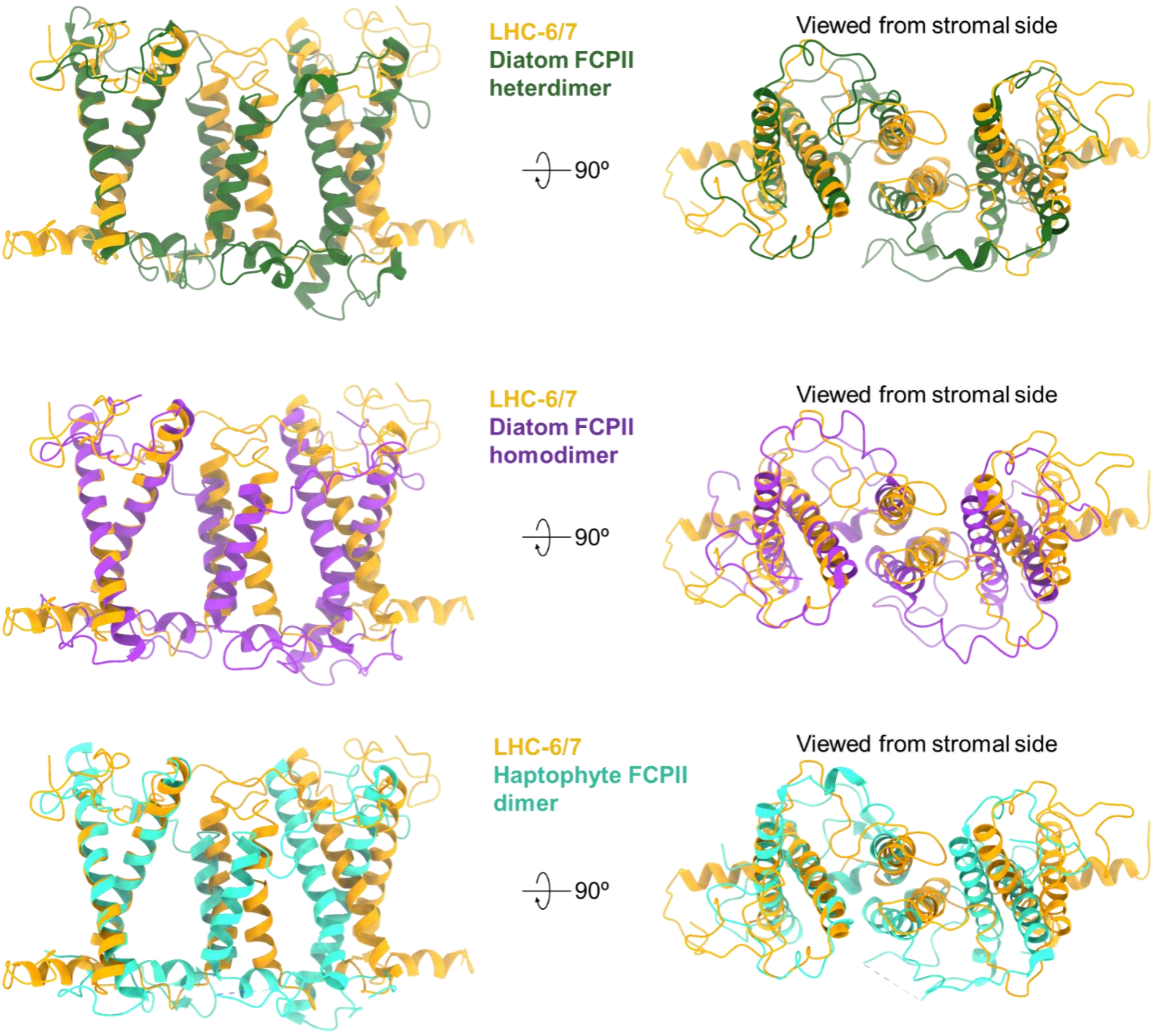

Supplementary Figure 11. Structural comparison of the euglenophyte LhcE dimer (orange) with diatom LHCII heterodimer (green) and homodimer (purple) (PDB: 8IWH) and haptophyte LHCII dimer (turquoise) (PDB: 9KQB).

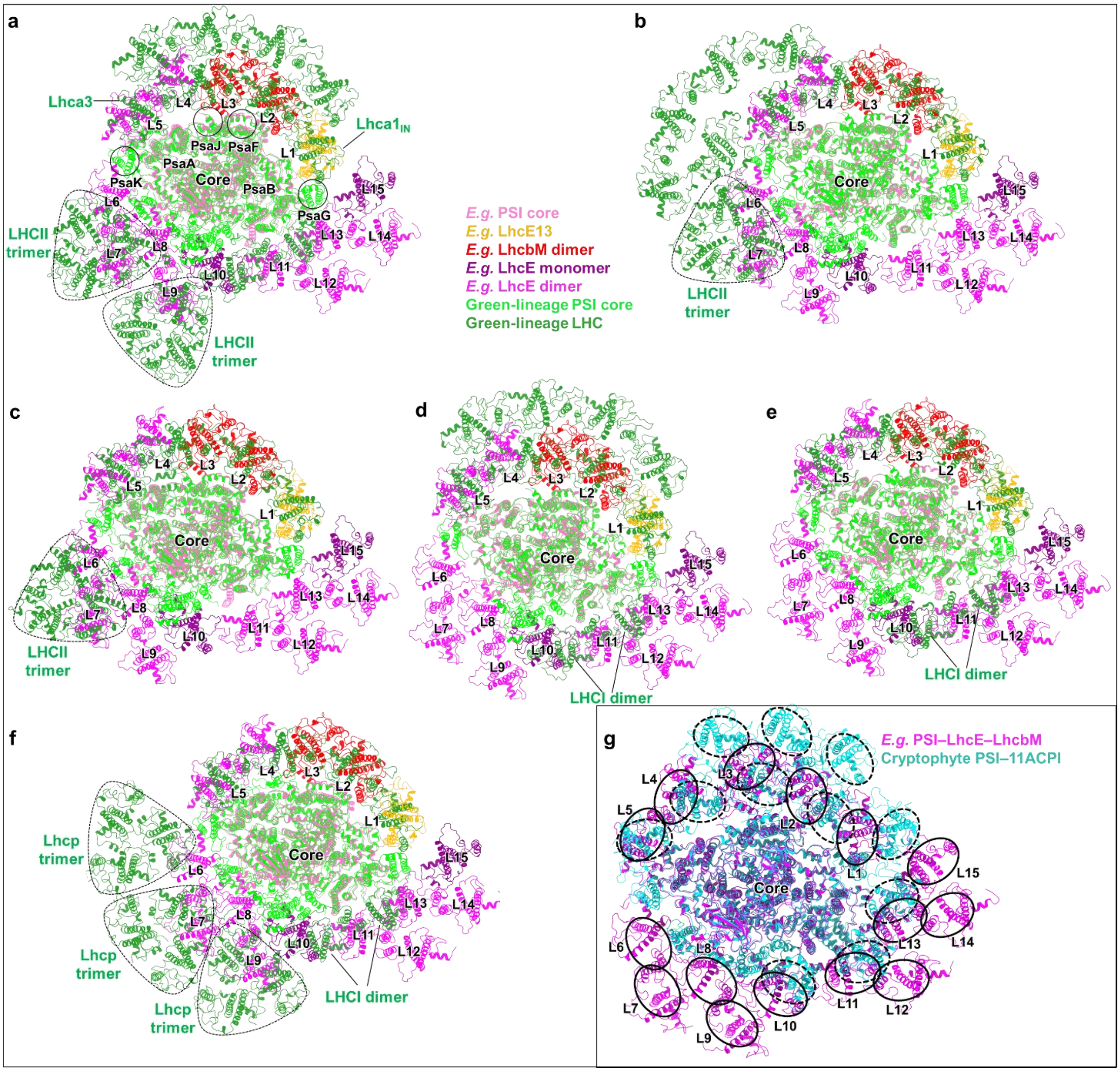

Supplementary Figure 12. Comparison of the arrangements of LHCs in euglenophyte PSI–LhcE–LhcbM with those in green-algal PSI–LHCs and cryptophyte PSI–ACPI. Superposition of the *E. gracilis* (*E.g.*) PSI–LhcE–LhcbM with PSI–LHCI–LHCII from green alga *Chlamydomonas reinhardtii* (PDB: 7DZ7) (**a**), moss *Physcomitrium patens* (PDB: 7XQP) (**b**), and land plant *Zea mays* (PDB: 5ZJI) (**c**), PSI–LHCI from green algae *Bryopsis corticulans* (PDB: 6IJO) (**d**) and *Dunaliella Salina* (PDB: 6SL5) (**e**), PSI–LHCI–Lhcp from *Ostreococcus tauri* (PDB: 7YCA) (**f**), and cryptophyte PSI–11ACPI (PDB: 7Y8A) (**g**). L1-L15 indicate 15 LHCs around the *E. gracilis* PSI core. Lhca1$_{IN}$, Lhca3, PsaA, PsaB, PsaF, PsaJ, PsaK, and PsaG in *Chlamydomonas reinhardtii* PSI–LHCI, LHCII trimer in green-lineage PSI–LHCI–LHCIIs, LHCI dimer in green algal PSI–LHCIs, and Lhcp trimer in *Ostreococcus tauri* PSI–LHCI–Lhcp are indicated. The locations and orientations of *E. gracilis* LHCs and cryptophyte ACPIs in panel **g** are indicated by solid circles and dashed circles, respectively.

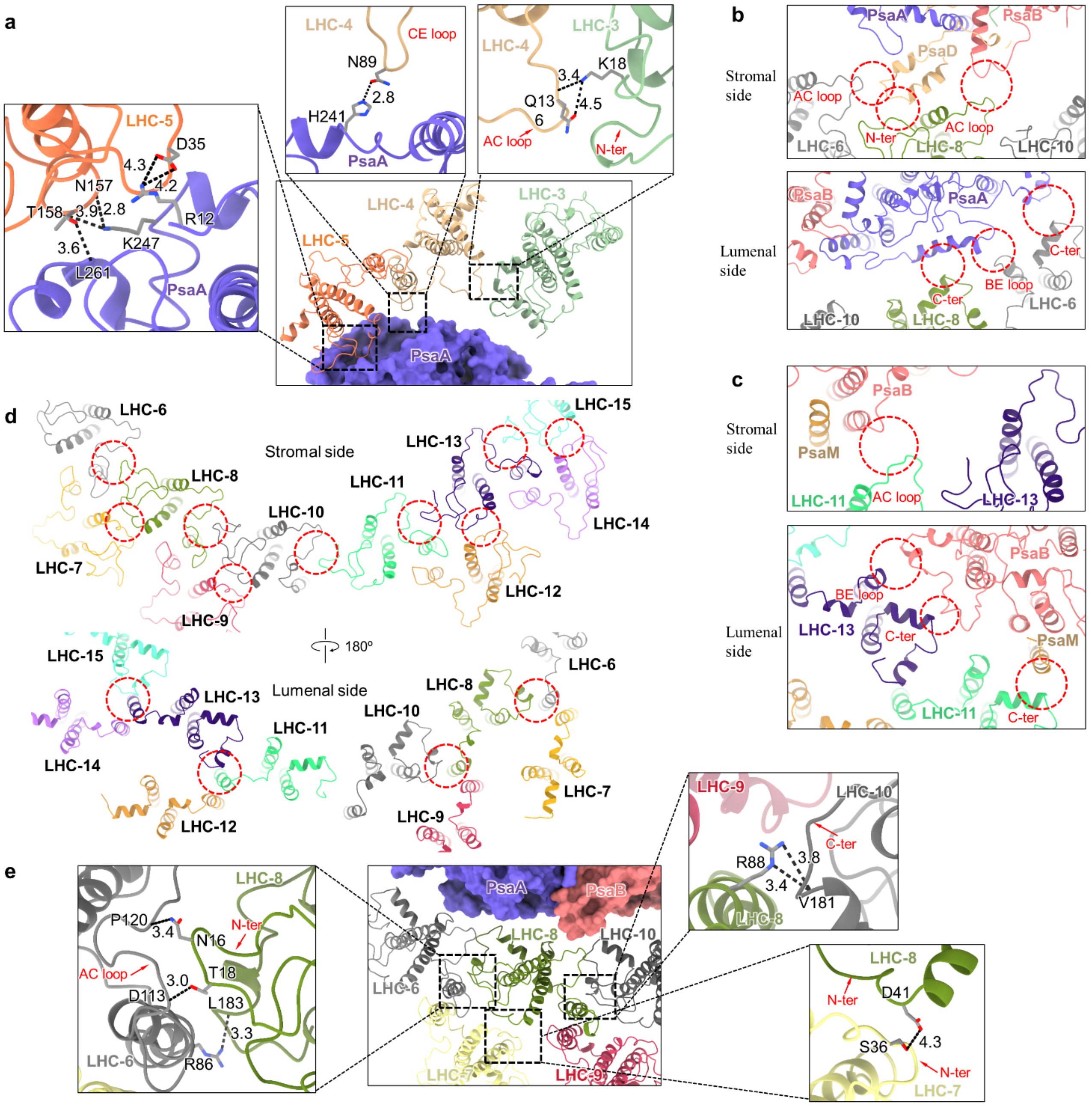

Supplementary Figure 13. Interactions between LHCs and the PSI core and between LHCs in euglenophyte PSI–LhcE–LhcbM.

a, Interactions between LHC-4/5 dimer with the PSI core and between LHC-4 with LHC-3. **b**, Interaction areas between LHC-6/8 with the PSI core. **c**, Interaction areas between LHC-11/13 with the PSI core. **d**, Interaction areas between the LHCs in LHC-6/7/8/9/10 module and LHC-11/12/13/14/15 module. **e**, Interactions between the LHCs in LHC-6/7/8/9/10 module. Interactions are indicated by black dashed lines with distances labeled in Å.

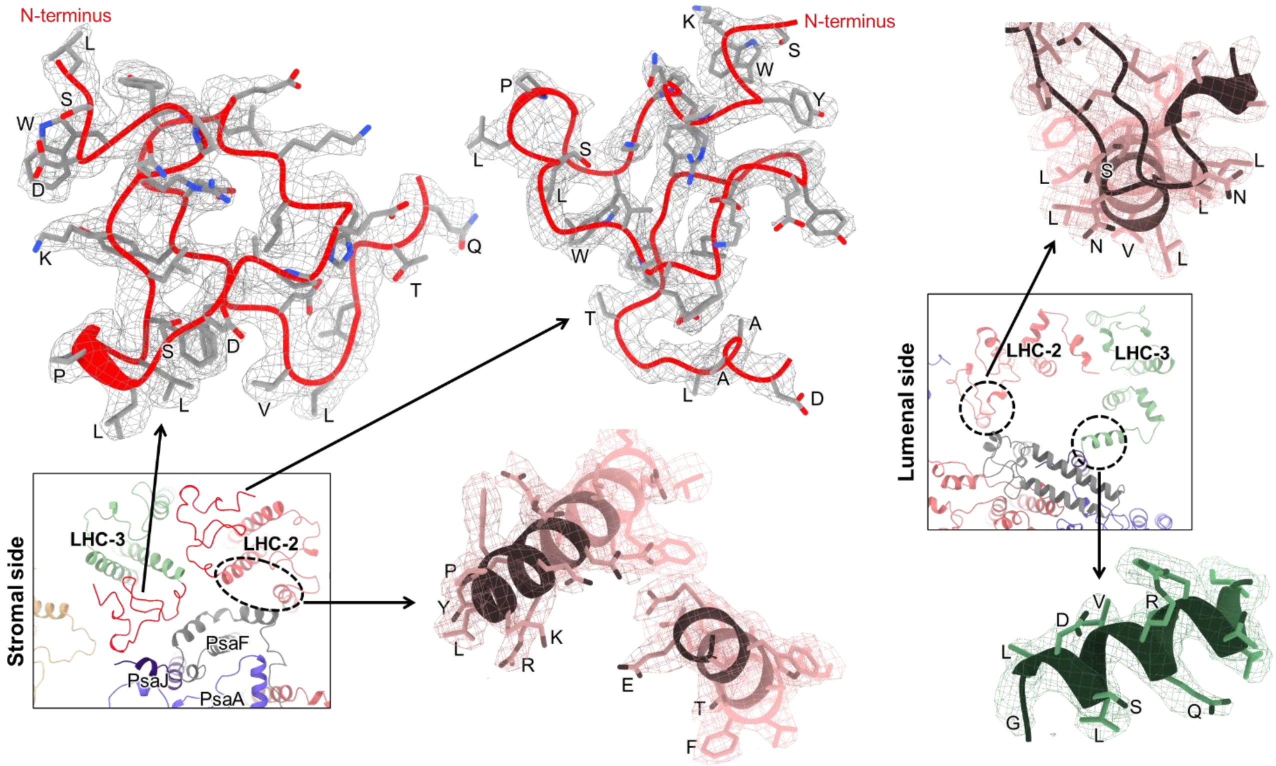

Supplementary Figure 14. Cryo-EM density maps of the N-termini within euglenophyte LhcbMs and structural domains which form interactions with the PSI core within euglenophyte LhcbMs. The N-terminus of LhcbM is colored red. Structural domains which form interactions with the PSI core are indicated by dashed circles. Residues at the interface between LhcbMs and the PSI core are indicated. RRpT motif and phosphorylated Thr residues were absent.

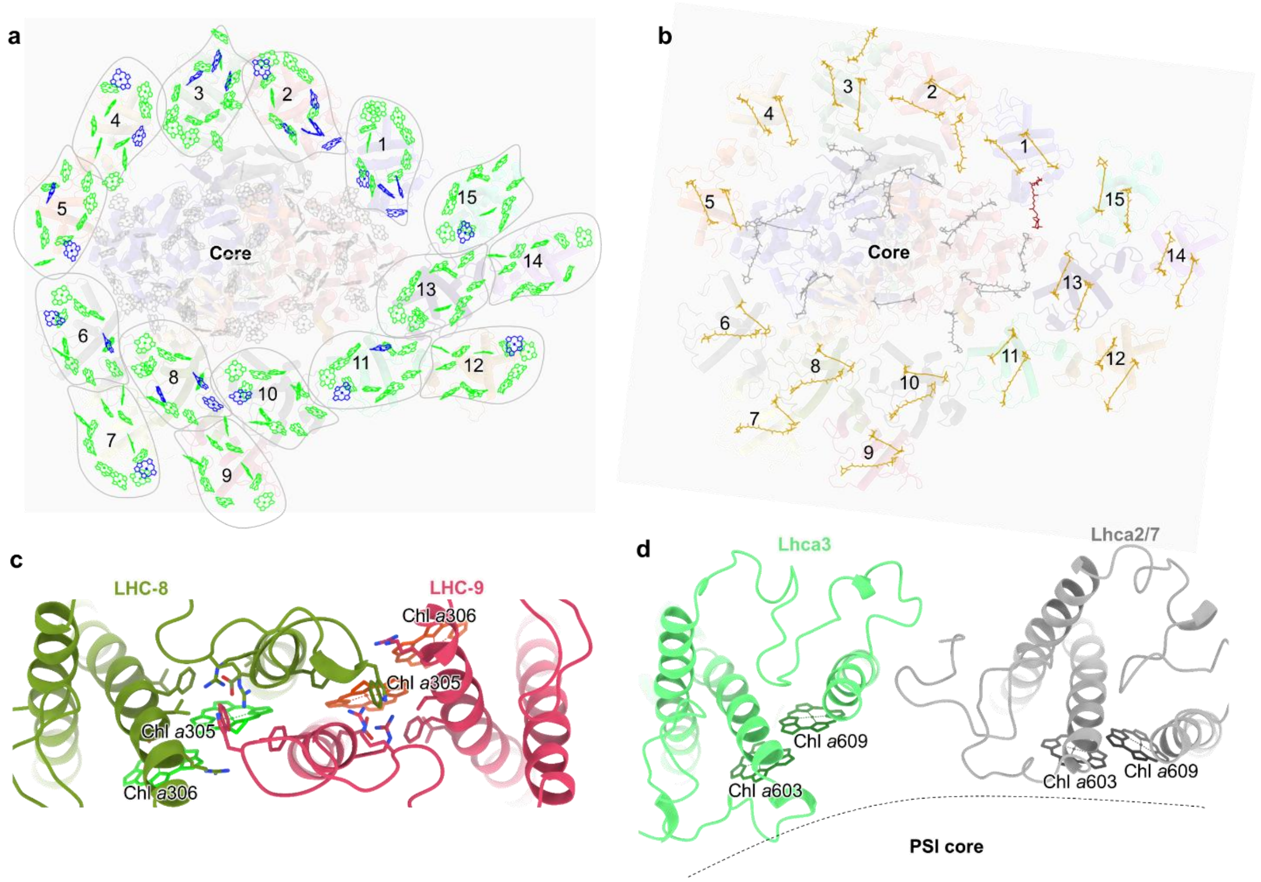

Supplementary Figure 15. Arrangement of the pigments in euglenophyte PSI–LhcE–LhcbM.
a, Arrangement of all Chls in euglenophyte PSI–LhcE–LhcbM. The core Chls are colored in gray. Chls in LHCs are colored in green (Chl *a*) and blue (Chl *b*). **b**, Arrangement of all Cars in euglenophyte PSI–LhcE–LhcbM. The core Cars are colored in gray. Cars in LHCs are colored in orange (Ddx) and brown (Neo). **c**, Arrangement pattern the two Chl *a*305/*a*306 pairs in centrosymmetric LhcE dimer and the amino acid residue environment surrounding the Chl *a*305/*a*306. **d**, Arrangement of Chl *a*603/*a*609 pairs in green algae and land plants.

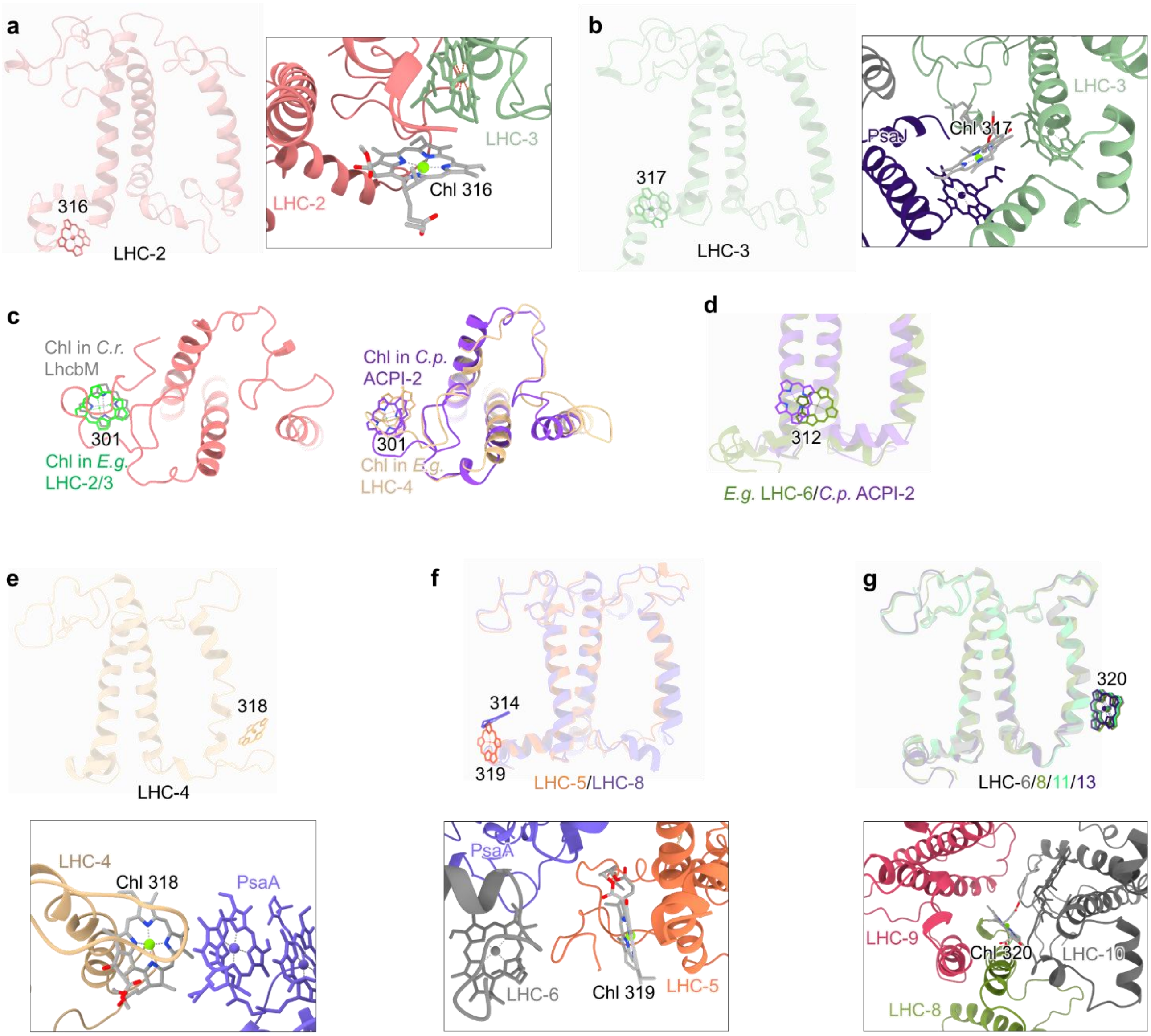

Supplementary Figure 16. Locations and orientations of pigments in euglenophyte LHCs.
a, **b**, The locations of Chl 316 and Chl 317 in euglenophyte LHCs. **c**, The locations and orientations of Chl 301 in euglenophyte LHCs of *E. gracilis* (*E.g.*) and comparation with those in green-algal LhcbM of *C. reinhardtii* (*C.r*) and in cryptophyte ACPI of *Chroomonas placoidea* (*C.p.*). **d**, The locations and orientations of Chl 312 in euglenophyte LHCs of *E. gracilis* (*E.g.*) and comparation with those in cryptophyte ACPI of *Chroomonas placoidea* (*C.p.*). **e-g**, The locations of Chl 314/318/319/320 in euglenophyte LHCs.

Supplementary Table 1. Cryo-EM data collection, refinement, and validation statistics.

	PSI–LhcE–LhcbM (EMDB-65121; PDB-9VJS)
**Data Collection and Processing**	
Voltage (kV)	300
Electron exposure (e- /Å2)	60
Defocus range (um)	-1.2~-2.2
Pixel size (Å)	0.53
Symmetry imposed	C1
Initial particle images (no.)	433,460
Final particle images (no.)	78,124
Map resolution (Å)	2.72
FSC threshold	0.143
**Refinement**	
Model composition	
Non-hydrogen atoms	54494
Protein residues	4734
Ligands	345
B factors (Å^2)	
Protein	26.23
Ligand	28.21
R.m.s. deviations	
Bond lengths (Å)	0.010
Bond angles (º)	1.907
Validation	
MolProbity score	1.37
Clashscore	3.32
Rotamer outliers (%)	0.11
Ramachandran plot	
Favored (%)	96.35
Allowed (%)	3.48
Disallowed (%)	0.17

Supplementary Table 2. Cofactors in each subunit of the euglenophyte PSI–LhcE–LhcbM supercomplex.

Subunits	Traced residues	Chls	Cars	Lipids	Others
PsaA	740 (12-751)	45 a	4 *β*-Car, 2 Ddx	1 PG	1 PQN, 1 SF4
PsaB	730 (4-733)	40 a	6 *β*-Car	1 MGDG, 1 DGDG,	1 PQN
PsaC	80 (2-81)				2 SF4
PsaD	187 (14-200)				
PsaE	63 (37-99)				
PsaF	166 (7-172)	3 a	1 *β*-Car, 1 Ddx		
PsaJ	37 (1-37)	1 a	1 β-Car, 2 Ddx		
PsaM	31 (1-31)		1 *β*-Car		
LHC-1	199 (9-207)	10 a, 4 b	2 Ddx, 1 Neo	2 MGDG	
LHC-2	219 (7-225)	10 a, 5 b	3 Ddx	1 MGDG	
LHC-3	222 (9-230)	12 a, 3 b	3 Ddx	1 MGDG	
LHC-4	172 (9-180)	9 a, 2 b	2 Ddx	1 MGDG	
LHC-5	166 (8-173)	9 a, 2 b	2 Ddx	1 MGDG	
LHC-6	172 (8-179)	11 a, 2 b	2 Ddx	1 MGDG	
LHC-7	168 (6-173)	10 a, 1 b	2 Ddx	1 MGDG	
LHC-8	182 (8-189)	10 a, 3 b	2 Ddx	2 MGDG	
LHC-9	167 (6-172)	9 a	2 Ddx		
LHC-10	178 (11-188)	11 a, 1 b	2 Ddx	1 MGDG	
LHC-11	172 (8-179)	11 a, 2 b	2 Ddx	1 MGDG	
LHC-12	168 (6-173)	10 a, 1 b	2 Ddx		
LHC-13	180 (10-189)	13 a	2 Ddx	1 MGDG	
LHC-14	156 (18-173)	9 a	2 Ddx		
LHC-15	177 (11-187)	11 a, 1 b	2 Ddx		
PSI–LhcE–LhcbM	4734	244 a, 27 b	13 *β*-Car, 37 Ddx, 1 Neo	14 MGDG, 1 PG, 1 DGDG,	3 SF4, 2 PQN

DGDG, digalactosyldiacyl glycerol; MGDG, monogalactosyldiacyl glycerol; PG, phosphatidyl glycerol; SQDG, sulfoquinovosyldiacyl glycerol; Chl c_2-MGDG, Chlorophyll c_2-monogalactosyldiacylglyceride; PQN, phylloquinone; SF4, sulphur–iron cluster.

Supplementary Table 3. The components of PSI core from euglenophyte, cyanobacteria, green algae, land plants, red algae, cryptophyte, diatom, haptophyte, dinoflagellate.

Subunit	Euglenophyte	Cyanobacteria	Green algae	Land plants	Red algae	Cryptophyte	Diatom	Haptophyte	Dinoflagellate
PsaA	●	●	●	●	●	●	●	●	●
PsaB	●	●	●	●	●	●	●	●	●
PsaC	●	●	●	●	●	●	●	●	●
PsaD	●	●	●	●	●	●	●	●	●
PsaE	●	●	●	●	●	●	●	●	●
PsaF	●	●	●	●	●	●	●	●	●
PsaG			●	●					
PsaH			●	●					
PsaI		●	●	●	●	●	●	●	●
PsaJ	●	●	●	●	●	●	●	●	●
PsaK		●	●	●	●	●		●	
PsaL		●	●	●	●	●	●	●	●
PsaM	●	●	●		●	●	●	●	●
PsaN			●	●					
PsaO			●	●	●	●			
PsaQ						●			
PsaR					●	●	●	●	●
PsaS							●		
PsaT									●
PsaU									●
PsaX		●							

●: present of the subunit.

Supplementary Table 4. Binding sites of pigments in the 15 LHC subunits of euglenophyte PSI–LhcE–LhcbM.

Sites	LHC-1	LHC-2	LHC-3	LHC-4	LHC-5	LHC-6	LHC-7	LHC-8	LHC-9	LHC-10	LHC-11	LHC-12	LHC-13	LHC-14	LHC-15
301	Chl *a*	Chl *b*	Chl *a*	Chl *b*	Chl *b*	Chl *b*	Chl *b*	Chl *a*		Chl *b*	Chl *b*	Chl *b*	Chl *a*		Chl *b*
302	Chl *a*	Chl *a*	Chl *a*	Chl *a*	Chl *a*	Chl *a*	Chl *a*	Chl *a*		Chl *a*	Chl *a*	Chl *a*	Chl *a*		Chl *a*
303	Chl *a*	Chl *a*	Chl *a*	Chl *a*	Chl *a*	Chl *a*	Chl *a*	Chl *a*	Chl *a*	Chl *a*	Chl *a*	Chl *a*	Chl *a*	Chl *a*	Chl *a*
304	Chl *a*	Chl *a*	Chl *b*	Chl *b*	Chl *b*	Chl *b*	Chl *a*	Chl *b*	Chl *a*	Chl *a*	Chl *b*	Chl *a*	Chl *a*	Chl *a*	Chl *a*
305	Chl *a*	Chl *a*	Chl *a*	Chl *a*	Chl *a*	Chl *a*	Chl *a*	Chl *a*	Chl *a*	Chl *a*	Chl *a*	Chl *a*	Chl *a*	Chl *a*	Chl *a*
306	Chl *a*	Chl *a*	Chl *a*	Chl *a*	Chl *a*	Chl *a*	Chl *a*	Chl *a*	Chl *a*	Chl *a*	Chl *a*	Chl *a*	Chl *a*	Chl *a*	Chl *a*
307	Chl *a*	Chl *a*	Chl *a*	Chl *a*	Chl *a*	Chl *a*	Chl *a*	Chl *b*	Chl *a*	Chl *a*	Chl *a*	Chl *a*	Chl *a*	Chl *a*	Chl *a*
308	Chl *a*	Chl *a*	Chl *a*	Chl *a*	Chl *a*	Chl *a*	Chl *a*	Chl *a*	Chl *a*	Chl *a*	Chl *a*	Chl *a*	Chl *a*	Chl *a*	Chl *a*
309	Chl *a*	Chl *a*	Chl *a*	Chl *a*	Chl *a*	Chl *a*	Chl *a*	Chl *a*	Chl *a*	Chl *a*	Chl *a*	Chl *a*	Chl *a*	Chl *a*	Chl *a*
310	Chl *b*	Chl *b*	Chl *a*												
311	Chl *b*	Chl *b*	Chl *b*												
312						Chl *a*		Chl *a*		Chl *a*	Chl *a*		Chl *a*		Chl *a*
313	Chl *b*	Chl *b*	Chl *b*	Chl *a*	Chl *a*	Chl *a*	Chl *a*	Chl *b*	Chl *a*	Chl *a*	Chl *a*	Chl *a*	Chl *a*	Chl *a*	Chl *a*
314	Chl *a*	Chl *a*	Chl *a*			Chl *a*	Chl *a*	Chl *a*	Chl *a*	Chl *a*	Chl *a*	Chl *a*	Chl *a*	Chl *a*	Chl *a*
315	Chl *b*	Chl *b*	Chl *a*												
316		Chl *a*													
317			Chl *a*												
318				Chl *a*											
319					Chl *a*										
320						Chl *a*		Chl *a*			Chl *a*		Chl *a*		
401	Ddx	Ddx	Ddx	Ddx	Ddx	Ddx	Ddx	Ddx	Ddx	Ddx	Ddx	Ddx	Ddx	Ddx	Ddx
402	Ddx	Ddx	Ddx	Ddx	Ddx	Ddx	Ddx	Ddx	Ddx	Ddx	Ddx	Ddx	Ddx	Ddx	Ddx
403	Neo	Ddx	Ddx												

Supplementary Table 5. The assigned sequence of each LHC and the corresponding accession number to each sequence in the National Center for Biotechnology Information.

Antenna	Sequence	NCBI accession number
LHC-1 (Chain a)	MLATSGEKRPTWFPGAEAPAWLTGEFPGDRGFDPCGLARDPVDFAKFRDSEVFHGRWA<br>MLGLVGCLVPEVFGNLGIAQLPAWYDAATVANTSNLDYLGNPNLIHASNVSFIALSTLLL<br>MGPVEAWRWNGALASEAKSAERTTYPGGPFDPLKLGASPELKLKEIKNGRLAMVGMF<br>GFWAQSYVTGEGPLANLAAHLADPAHNNLLNTVA	GDJR01039000.1
LHC-2 (Chain b)	MFAATGSKWYGPNRPKWLGPLSGGAVPEYLKGEYAGDYGFDTAGLAADPKLFQRYRD<br>AELQNGRWAMLGVLGCLAPEVLSNVFGVPYPEPVWFKTGATILNGGSIDYLANPKLIHA<br>SNLLLTLVLELVFFFAAETWREAGEGPLGKAQDKSYPGGVFDPLGLSKDPAAFAEAKVK<br>EVKNGRLAMLAMLGLFVQAGVTGQSPLENLSAHLANPGVNFWTSYAPTLA	DAA05881.1
LHC-3 (Chain c)	MFAATGEKLSDWYGPNRKLFLGPLSDGAPEHLKGELPGDYGFDVLGLATQPTRLERYR<br>QGEIINGRWAMLGIVGCIVPELLARNYGVPFPEPVWFKTGATVFSEEGLNYLGNPSLIHA<br>KSIAAILVTEILFVGAAEAFRVSGGPLGPATDLVYPGKAFDPLGLSKDETAFAELKVKEVK<br>NGRLAMLGMLGLFMQGFATGKGPLQNLADHLADPTGANIITFHQRVLSDLG	DAA05882.1
LHC-4 (Chain d)	MFTTTGEKRPVWFPGATPPAYLTGEFPADRGFDPLGLAKDPATYERMRSSEVFHSRLAML<br>GVVGSLIPELQGQGAWYTLSEKQFGPGPNGEVIGFTELALIGMLFSYPLEYWRGNGGFG<br>WFEEKGDRTYPGFDPLQLTSEYTKTAEIKNGRLAMSALLGFAVQHATTGASPLENWAAT<br>TGGSVA	ABW06953.1
LHC-5 (Chain e)	MFAAAGERATWFPGAKAPPHLTGEFPGDRGFDPVDFAANPESFERMRASEVFHGRMAM<br>LGVAGCLIPELLGRGVWFQAGESVDAAQLGLYFMFLAAPSEYWRGNGGFGWDQKEKK<br>AGNRIYPGFDPLKMTSDETKTKEIKNGRLAMIAFLGLVSQANTTGVSPFQNLAAVFGASP<br>VA	ABW06952.1
LHC-6 (Chain f)	MAASSGERLLWIPNATPPAHLTGEFPGDRGFDPLGLSKDPKVFARMRISEVFHGRLAMLG<br>IVGCVGQEWLFNKGAWFDYSDFDLPRLGLIALQVIAPLEYWRGNGGFSWNGNDGPDRS<br>YPGFDPLGLTTEDTKLREIKNGRLAMSAMLGLEVQSHITGKSPLTNLGDHLSSPFTANILT<br>GGGSVA	ABW06947.1
LHC-7/9/12/14 (Chain k/l/m/n)	MFAATGHKDGVWFPGATPPAHLTGEYPADRGFDPLSLAADPTVYARMRVSEVFHARLS<br>MLAIVGAIVPEVLGKGAWFEAGNSVDGIKLGFIAMAIAAPTEYWRGNGGFNWDKGAA<br>DRSYPGFDPLKLTTDYTKAAEIKNGRLAMAGLLGLTFQYLATGESPLANLAAHLANPVG	ABW06948.1
LHC-8/13 (Chain g/j)	MYSTSGEKYRGLWFPNITPPAYLTGEFPADRGFDPAGLAADPKVYERMRIAEVFNGRLA<br>MLAIVGCVYPELLGNGVWFEVWNKVDFYRFALISLQVVAPLEYWRGNGGFGWDGEEK<br>YDRSYPGFDPCNLTTEYTKAAEIKNGRLAMIGMFGLEVQSHVTAQGPVANLIEHLRHPL<br>AANIGANLAHPWPPVA	ABW06948.1
LHC-10/15 (Chain h/o)	MFSTAGQKTDRRPLWFPGGYAPSWLTGQHFGDRGFDPAGLAADPKVFERMRVSEVYHG<br>RLAMLAIVGAVVPDIQGKGAWYEAAQSAGIGVNEVAVFTAAYGIAEVARGLKANSDPTS<br>RYSGFDSLNLTTDYTKEAEIKNGRLALTAMLGLQVQRHVTGVSPLLNLVEHVKHPLVHTI<br>AESVMHQWPVA	ABW06949.1
LHC-11 (Chain i)	MAATTGERLLWIPNATPPAHLTGEFPGDRGFDPLGLAKDPKVYQRMRISEVFHGRLAML<br>GIVGCVAPELFFSKGAWFDYSDYDLNRLGLIALQVIAPLEYWRGNGGFSWDGNDGPDRS<br>YPGFDPLGLTNEETKLQEIKNGRLAMTAMLGLEVQSHITGKSPLTNLSEHLSHPLSANLL<br>TGGGSLA	ABW06949.1

Supplementary Table 6. Chl *b* assignment in LHC subunits of euglenophyte PSI–LhcE–LhcbM.

Antenna	Chl *b* Sites	Evidence supporting Chl *b* assignment			
		Map density of the C7-formyl group	Hydrogen bonding of C7-formyl group with charged residues or polar residues	Sequence conservation of the binding site	Refer to counterparts in green algal LhcbM
LHC-1	310	√			√
	311	√	√		√
	313	√		√	√
	315	√		√	√
LHC-2	301	√		√	√
	310	√			√
	311	√	√		√
	313	√		√	√
	315	√		√	√
LHC-3	304	√			
	311	√	√		√
	313	√		√	√
LHC-4	301			√	
	304	√			
LHC-5	301	√		√	
	304	√			
LHC-6	301	√		√	
	304	√			
LHC-7	301			√	
LHC-8	304	√			
	307	√			
	313	√			
LHC-10	301	√		√	
LHC-11	301			√	
	304	√			
LHC-12	301			√	
LHC-15	301			√	

Reference:

1. Rory J Craig, Sean D Gallaher, Shengqiang Shu, Patrice A Salomé, Jerry W Jenkins, Crysten E Blaby-Haas, Samuel O Purvine, Samuel O'Donnell, Kerrie Barry, Jane Grimwood, Daniela Strenkert, Janette Kropat, Chris Daum, Yuko Yoshinaga, David M Goodstein, Olivier Vallon, Jeremy Schmutz, and Sabeeha S Merchant, The Chlamydomonas Genome Project, version 6: Reference assemblies for mating-type plus and minus strains reveal extensive structural mutation in the laboratory. Plant Cell 35, 644-672, doi:10.1093/plcell/koac347 (2023).

2. Philippe Lamesch, Tanya Z. Berardini, Donghui Li, David Swarbreck, Christopher Wilks, Rajkumar Sasidharan, Robert Muller, Kate Dreher, Debbie L. Alexander, Margarita Garcia-Hernandez, Athikkattuvalasu S. Karthikeyan, Cynthia H. Lee, William D. Nelson, Larry Ploetz, Shanker Singh, April Wensel, and Eva Huala, The Arabidopsis Information Resource (TAIR): improved gene annotation and new tools. Nucleic Acids Res 40, D1202-1210, doi:10.1093/nar/gkr1090 (2012).

3. David M. Goodstein, Shengqiang Shu, Russell Howson, Rochak Neupane, Richard D. Hayes, Joni Fazo, Therese Mitros, William Dirks, Uffe Hellsten, Nicholas Putnam, and Daniel S. Rokhsar, Phytozome: a comparative platform for green plant genomics, Nucleic Acids Res. 2012 40 (D1): D1178-D1186, doi:10.1093/nar/gkr944 (2012).
